# The hidden genome of high-grade serous ovarian carcinoma: repetitive DNA, extrachromosomal DNA, and complex structural variation

**DOI:** 10.3389/fmolb.2026.1988095

**Published:** 2026-10-28

**Authors:** Xinhua Xu, Xiaochen Ren, Zeng Yuan

**Affiliations:** 1 Department of Obstetrics and Gynecology, Zibo 148 Hospital, China RongTong Medical Healthcare Group Co. Ltd, Zibo, Shandong, China; 2 Department of Obstetrics and Gynecology, Qilu Hospital of Shandong University, Jinan, Shandong, China

**Keywords:** extrachromosomal DNA, genomic instability, high-grade serous ovarian carcinoma, long-read sequencing, repetitive DNA, structural variation, transposable elements

## Abstract

High-grade serous ovarian carcinoma (HGSOC) is marked by extensive genomic instability, characterized by near-universal *TP53* mutations, frequent homologous recombination deficiency (HRD), widespread copy-number alterations, and complex structural rearrangements. However, much of our current understanding of this molecular landscape has been derived from exome sequencing, microarrays, and short-read whole-genome sequencing, which are limited in their ability to resolve repetitive and structurally complex genomic regions. Long-read sequencing, together with telomere-to-telomere reference assemblies, is beginning to reveal previously inaccessible aspects of HGSOC genome architecture. Exploratory studies have reported centromeric and transposable-element hypomethylation, chromosome-arm-specific telomere shortening, and complex extrachromosomal DNA (ecDNA) architectures associated with enhancer regulation and transposable-element activity. Complementary studies have also identified ongoing LINE-1 retrotransposition in HGSOC. Together, these observations suggest that repetitive DNA, epigenetic instability, and complex genome architecture may contribute to HGSOC heterogeneity and tumor evolution, although their causal relationships remain uncertain. Nevertheless, genome-wide long-read evidence in HGSOC currently derives primarily from two small studies involving six and 12 patients, respectively, underscoring the need for validation in larger, independent cohorts.

## 1 Introduction

High-grade serous ovarian carcinoma (HGSOC) is a paradigmatic example of a genomically unstable malignancy. The Cancer Genome Atlas established a molecular framework characterized by near-universal *TP53* mutations, extensive copy-number alterations, and homologous recombination deficiency (HRD) in approximately half of all cases (Cancer Genome Atlas Research Network, 2011). Subsequent whole-genome analyses have further revealed that structural rearrangements play a central role in driving tumor suppressor loss, oncogene amplification, and therapeutic resistance. Notably, gene-disrupting rearrangements affecting *RB1*, *NF1*, *RAD51B*, and *PTEN*, amplification of *CCNE1*, and *BRCA1/2* reversion events have all been linked to the emergence of chemoresistant disease (Patch et al., 2015). More recently, copy-number and rearrangement signatures have offered a more systematic understanding of the mutational processes shaping the seemingly chaotic HGSOC genome (Hillman et al., 2018; Macintyre et al., 2018).

Yet this framework relies largely on exome sequencing, microarrays, or short-read whole-genome sequencing. These methods detect point mutations and copy-number alterations but poorly resolve repeats, complex breakpoints, centromeres, telomeres, and circular DNA. Moreover, they offer limited capacity to phase genetic and epigenetic changes along individual DNA molecules (Nurk et al., 2022; Li et al., 2025). Consequently, repetitive and structurally intricate regions remain underrepresented in cancer genome analyses. In this Mini Review, we use the term “hidden genome” to refer broadly to repetitive and structurally complex genomic compartments that have historically been incompletely resolved by conventional sequencing approaches.

Long-read sequencing can span repetitive regions and complex rearrangement junctions, while nanopore sequencing also enables direct assessment of DNA methylation. The telomere-to-telomere (T2T) human reference assembly provides a complementary advance, incorporating nearly 200 Mb of sequence absent from earlier reference genomes, including centromeric satellite arrays (Nurk et al., 2022). Together, these developments enable more detailed investigation of genomic regions that have remained difficult to characterize in HGSOC. This Mini Review examines emerging evidence concerning centromeres, telomeres, transposable elements (TEs), extrachromosomal DNA (ecDNA), and complex structural variation in HGSOC. The principal features and hypothesized relationships are summarized in Figure 1.

**FIGURE 1:**
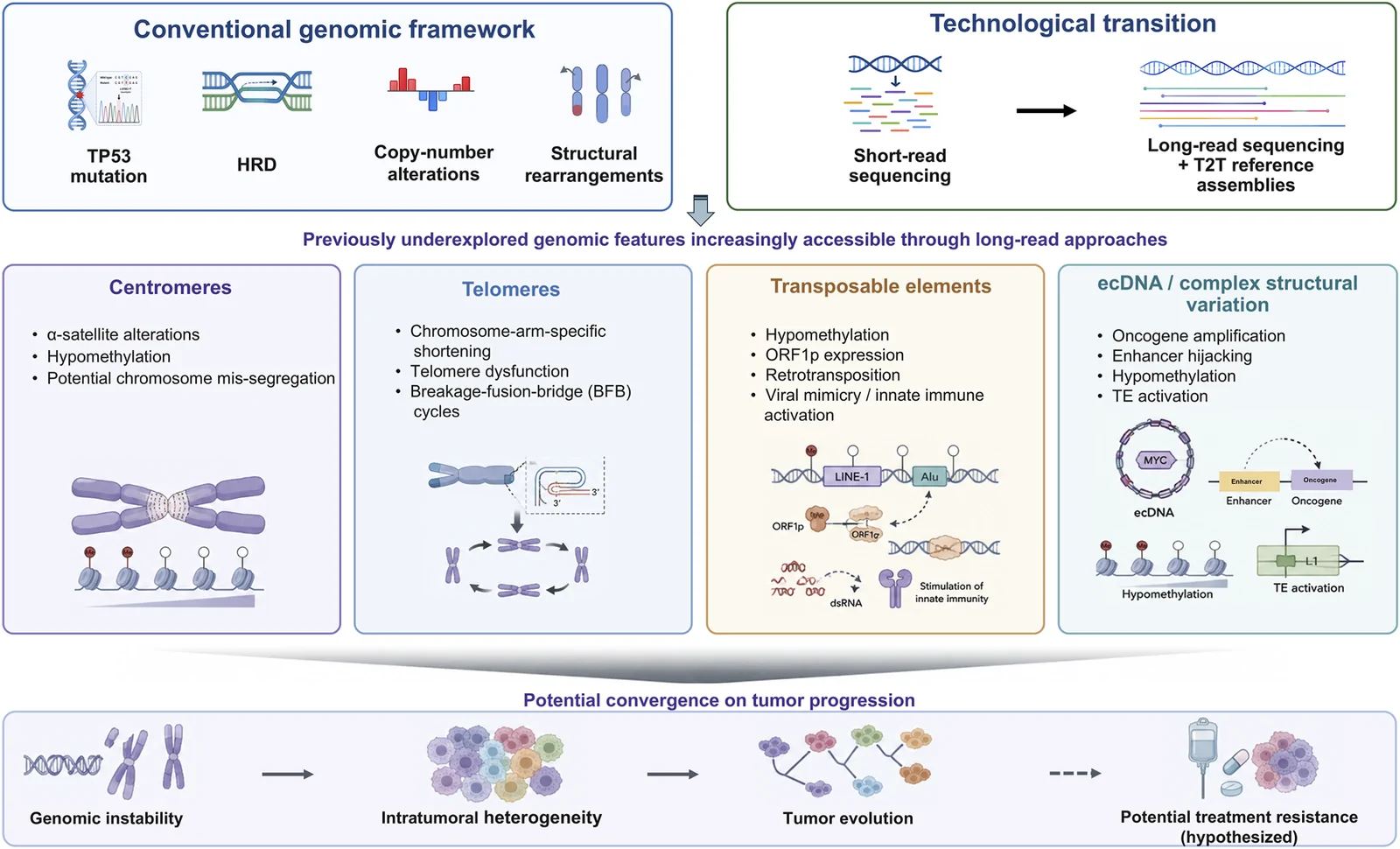
From the conventional to the hidden genome of high-grade serous ovarian carcinoma. The conventional genomic framework of high-grade serous ovarian carcinoma (HGSOC) is characterized by near-universal TP53 mutation, homologous recombination deficiency (HRD), widespread copy-number alterations, and complex structural rearrangements. Long-read sequencing, together with telomere-to-telomere (T2T) reference assemblies, extends this framework by enabling improved interrogation of repetitive and structurally complex genomic regions that are incompletely resolved by short-read approaches. Emerging features include centromeric α-satellite alterations and hypomethylation, chromosome-arm-specific telomere shortening and its potential relationship to breakage-fusion-bridge (BFB) cycles, transposable-element derepression and LINE-1 retrotransposition, and extrachromosomal DNA (ecDNA) associated with oncogene amplification, enhancer hijacking, hypomethylation, and transposable-element activation. Collectively, these previously underexplored components of genome architecture may contribute to genomic instability, intratumoral heterogeneity, and tumor evolution. Their potential contribution to treatment resistance in HGSOC remains hypothetical and requires further validation.

## 2 From genomic chaos to previously inaccessible genome architecture

### 2.1 What conventional sequencing has already taught us

HGSOC genomic instability follows recurrent patterns. Copy-number signature analyses have uncovered seven reproducible patterns that reflect distinct mutational processes, revealing that individual tumors arise from a combination of these mechanisms (Macintyre et al., 2018). Likewise, rearrangement signatures have highlighted recurring structural patterns with potential clinical relevance (Hillman et al., 2018). Deep whole-genome sequencing of 324 HGSOC tumors further characterized this complexity. Ewing et al. identified frequent HRD and whole-genome duplication (WGD), alongside chromothripsis, breakage-fusion-bridge (BFB) events, chromoplexy, and ecDNA. Notably, two broad evolutionary trajectories emerged: one characterized by HRD and deletion-rich genomes, and another associated with WGD and an enrichment of duplications, chromothripsis, BFB events, and ecDNA (Ewing et al., 2025). These findings underscore that the extreme structural instability seen in HGSOC cannot be attributed to HRD alone.

Structural alterations thus have considerable biological relevance. In the 324-tumor cohort, somatic structural variants and copy-number alterations dominated the genomic landscape, with ecDNA predicted in a subset of tumors, some of which carried amplified oncogenes (Ewing et al., 2025). Earlier targeted insertion profiling had also identified somatic LINE-1 insertions in ovarian serous carcinoma (Tang et al., 2017). This raises a critical question: if conventional whole-genome sequencing already reveals such remarkable structural diversity, what additional processes remain hidden—those occurring within repetitive regions or requiring reconstruction of long-range molecular architecture?

### 2.2 What remained hidden

Short reads poorly map repetitive sequences and may fragment complex rearrangements into disconnected breakpoint calls. In contrast, long reads can directly span many of these regions and connect structural variants across tens of kilobases or more. Nanopore sequencing further enables simultaneous analysis of sequence and methylation on individual DNA molecules, allowing allele- or haplotype-resolved epigenomic profiling (Li et al., 2025). The T2T-CHM13 assembly offers a complementary reference framework by resolving previously incomplete centromeres, segmental duplications, and other repetitive regions (Nurk et al., 2022).

Small long-read studies have reported alterations in centromeric satellite DNA, telomere architecture, transposable elements, and extrachromosomal DNA that were difficult to fully characterize with earlier methods (Sun et al., 2025; Takamatsu et al., 2025). These findings suggest that previously inaccessible regions may influence HGSOC evolution. Key studies underpinning this emerging framework are summarized in Table 1.

**TABLE 1:** Key studies shaping the emerging hidden-genome framework of HGSOC.

Study	Samples/Model	Approach	Genome feature	Main finding	Relevance
Cancer Genome Atlas Research Network (2011)	489 HGSOCs (WES, n = 316)	Expression, CNA, methylation; WES	Conventional genome	*TP53* mutated in 96%; homologous recombination defects in ∼50%; widespread CNA	Established the molecular framework of HGSOC
Patch et al. (2015)	92 patients	Tumor/germline WGS	Structural variation	*RB1*/*NF1*/*RAD51B*/*PTEN* breakage; *CCNE1* amplification; *BRCA* reversions linked to resistance	Defined structural mechanisms of chemoresistance
Macintyre et al. (2018)	117 HGSOCs; validation in 527 cases	sWGS	Copy-number signatures	Seven reproducible CN signatures reflecting distinct mutational processes	Deconvoluted copy-number instability
Hillman et al. (2018)	80 AOCS HGSOCs + 490 TCGA cases	WGS; TCGA CNA validation	Rearrangement signatures	Five HGSOC rearrangement signatures; Ov.RS3 associated with poorer OS	Linked SV patterns with clinical outcome
Pisanic et al. (2019)	12 p53 signatures; 32 STICs; 112 ovarian carcinomas	ORF1p IHC; LINE-1 promoter methylation	LINE-1	LINE-1 ORF1p expression is more frequent in STICs than in p53 signatures	Supports possible early LINE-1 derepression without establishing its precise timing
Cen et al. (2022)	4 paired primary/metastatic HGSOCs	Circle-seq	eccDNA	Abundant eccDNA with distinct profiles in primary and metastatic tumors	Early evidence of circular-DNA heterogeneity
Sato et al. (2023)	30 HGSOCs (18 with STIC) + 12 benign FT + 6 incidental STICs	IHC; targeted MS; cell models	LINE-1 ORF1p	ORF1p detected in STIC/HGSOC and in ascites/plasma; absent from benign FT	Supports ORF1p as an early-event biomarker candidate
Mombach et al. (2024)	Two ovarian cancer cell-line models	RNA-seq; ATAC-seq; bioinformatics	TEs	Cisplatin alters TE transcription; TEs associate with host-gene regulation and chimeric transcripts	Shows TE functions beyond insertional mutagenesis
Ishak et al. (2025)	Premalignant FT lesions + syngeneic EOC models	Multiomic and functional analyses	Repetitive elements	p53 loss permits repetitive-element transcription and chronic viral mimicry, reducing immunogenicity	Links repetitive-element activity to early immune adaptation
Ewing et al. (2025)	324 HGSOCs	Deep WGS	Complex SV/ecDNA	Two structural trajectories: HRD-associated vs. WGD-dominated with frequent chromothripsis, BFB and ecDNA	Defines major structural evolutionary contexts
Takamatsu et al. (2025)	6 pretreatment HGSOCs + matched blood	Nanopore LRS + T2T-CHM13	Centromeres, TEs, telomeres	Centromeric/TE hypomethylation, HRD-related methylation patterns and chromosome-arm telomere shortening	Directly interrogates previously inaccessible repetitive regions
Sun et al. (2025)	24 primary/paired metastatic tumors from 12 HGSOC patients + 6 normal FT	Long-read WGS	ecDNA	ecDNA hypomethylation, enhancer rewiring/hijacking and TE activation	Connects ecDNA structure, epigenetics and TE activity
Pradhan et al. (2026)	Cohort 1: 50 tumors/28 patients; cohort 2: 57 tumors/38 patients	WGS/xTea; LDI-PCR; nanopore sequencing	LINE-1 insertions	Ongoing *de novo* LINE-1 retrotransposition contributes to site-to-site intrapatient heterogeneity	Supports ongoing LINE-1–mediated genome evolution

Abbreviations: AOCS, Australian Ovarian Cancer Study; ATAC-seq, assay for transposase-accessible chromatin using sequencing; BFB, breakage-fusion-bridge; CNA, copy-number alteration; CN, copy number; eccDNA, extrachromosomal circular DNA; ecDNA, extrachromosomal DNA; EOC, epithelial ovarian cancer; FT, fallopian tube; HGSOC, high-grade serous ovarian carcinoma; HRD, homologous recombination deficiency; IHC, immunohistochemistry; LDI-PCR, long-distance inverse PCR; LINE-1, long interspersed nuclear element-1; LRS, long-read sequencing; MS, mass spectrometry; ORF1p, open reading frame 1 protein; OS, overall survival; RNA-seq, RNA sequencing; STIC, serous tubal intraepithelial carcinoma; SV, structural variation; sWGS, shallow whole-genome sequencing; T2T, telomere-to-telomere; TCGA, the cancer genome atlas; TE, transposable element; WES, whole-exome sequencing; WGD, whole-genome duplication; WGS, whole-genome sequencing.

## 3 Centromeres and telomeres: overlooked components of chromosomal instability

### 3.1 Centromeric epigenetic instability

Human centromeres are composed largely of megabase-scale arrays of α-satellite DNA, organized within a specialized chromatin environment that facilitates CENP-A deposition, kinetochore assembly, and accurate chromosome segregation (Black and Giunta, 2018; Scelfo and Fachinetti, 2019). Their repetitive architecture complicates assembly and analysis. DNA methylation represents one layer of centromeric chromatin regulation, and both experimental and observational studies in various contexts have associated disruption of centromeric or pericentromeric methylation with aberrant satellite transcription, recombination, chromosome mis-segregation, and aneuploidy (Black and Giunta, 2018; Scelfo and Fachinetti, 2019).

In a recent study, Takamatsu et al. applied Oxford Nanopore sequencing and the T2T-CHM13 reference to six primary HGSOC tumors and matched blood samples (Takamatsu et al., 2025). Compared with matched blood, all tumors exhibited pronounced hypomethylation across centromeric satellite regions. Furthermore, centromeric methylation profiles distinguished tumors with high versus low HRD scores, with more substantial hypomethylation observed in the low-HRD group. The study also noted reduced or variable α-satellite read coverage in tumors, pointing to additional structural heterogeneity within centromeric arrays.

Interpretation is limited by the small cohort and the absence of tissue-matched normal fallopian tube controls. As such, the results do not establish a causal link between centromeric hypomethylation and chromosomal instability in HGSOC. Rather, they suggest that centromeric epigenetic instability may represent an additional facet of the chromosomal instability phenotype, and that its interplay with HRD could be more nuanced than previously recognized (Takamatsu et al., 2025).

### 3.2 Telomere architecture

Telomere dysfunction represents another mechanistic link between repetitive DNA and structural genomic instability. Critically shortened or dysfunctional telomeres can undergo end-to-end fusions, resulting in dicentric chromosomes that give rise to chromosome bridges and BFB cycles. BFB cycles can generate amplifications, deletions, translocations, and chromothripsis (Maciejowski and de Lange, 2017).

Long-read sequencing of HGSOC revealed pronounced telomere shortening at the level of individual chromosome arms and identified allele-specific methylation of the *TERT* hypermethylated oncological region (THOR) in three of six tumors (Takamatsu et al., 2025). Such allele-specific epigenetic alterations are difficult to detect using conventional bulk methylation assays. These findings do not establish a telomere-driven HGSOC subtype but suggest links between telomere architecture, epigenetic regulation and structural instability. This connection is particularly relevant given the enrichment of BFB cycles and chromothripsis in structurally unstable HGSOC subgroups (Ewing et al., 2025).

## 4 Transposable elements: from silent repeats to active genome modifiers

### 4.1 Epigenetic derepression during HGSOC development

Transposable elements constitute a substantial portion of the human genome and are typically kept in check by DNA methylation and other epigenetic mechanisms. Among them, LINE-1 (L1) is of particular interest, as it represents the only autonomous, protein-coding retrotransposon family still active in humans. A small subset of full-length L1 loci encodes ORF1p and ORF2p, which together facilitate retrotransposition via an RNA intermediate (Mendez-Dorantes and Burns, 2023). Consequently, the loss of epigenetic repression can transform repetitive DNA from a largely silent genomic compartment into a source of transcriptional and structural variation.

Pisanic et al. investigated L1 ORF1p expression in p53 signatures, serous tubal intraepithelial carcinomas (STICs), and ovarian carcinomas. None of the 12 p53 signatures showed substantial expression, whereas strong expression was frequently observed in STICs and correlated with L1 promoter hypomethylation. L1 expression also remained prevalent in invasive ovarian carcinomas (Pisanic et al., 2019). These observations suggest that L1 derepression may occur during early serous carcinogenesis, although the cross-sectional nature of the study precludes definitive conclusions about its timing or sequence during tumor development.

Sato et al. further demonstrated that ORF1p is expressed in HGSOC and tubal precursors, but not in benign fallopian tube epithelium, and can be released into conditioned medium, ascites, and plasma (Sato et al., 2023). A recent long-read sequencing study by Takamatsu et al. adds an epigenomic dimension to these observations, revealing pronounced hypomethylation of L1HS and endogenous retroviral elements in HGSOC, whereas AluY and SVA elements exhibited more modest changes (Takamatsu et al., 2025).

### 4.2 Retrotransposition as a continuing source of genome evolution

Active L1 elements can also reshape the genome. Pan-cancer whole-genome analyses have shown that somatic L1 retrotransposition can lead to insertions, large deletions, translocations, duplications, and even trigger BFB-associated oncogene amplification (Rodriguez-Martin et al., 2020). Beyond the general genomic instability of HGSOC, site-specific *de novo* L1 insertions provide direct evidence of retrotransposition during advanced disease (Pradhan et al., 2026).

Pradhan et al. examined *de novo* L1 insertions in clinical HGSOC samples and observed considerable inter-patient variability in L1 burden. Among L1-high patients, within-patient tumor pairs showed less overlap of L1 insertion sites than of single-nucleotide variants, quantified separately using the Jaccard index (shared events divided by the union of events). Given differences in variant abundance and detection, this comparison is descriptive rather than a measure of equivalent mutation rates. The site-specific insertions support ongoing retrotransposition and intrapatient heterogeneity (Pradhan et al., 2026). Tumors with high L1 burden also displayed proliferative characteristics, whereas those with little or no detectable insertion activity were comparatively enriched for immune response and cell death pathways.

Active L1 elements may therefore contribute to structural variation and genomic plasticity. Whether this activity is actively selected during tumor evolution or primarily reflects broader epigenetic dysregulation remains an open question.

### 4.3 Beyond insertional mutagenesis

Transposable element (TE) activation also has non-insertional consequences. Transcriptomic and chromatin profiling in ovarian cancer models has revealed that cisplatin exposure and resistance are associated with altered expression of LINEs, Alu elements, and endogenous retroviruses. TE transcription often correlates with the expression of nearby host genes and can give rise to chimeric TE–gene transcripts, offering additional mechanisms through which repetitive sequences may influence cellular programs (Mombach et al., 2024).

In addition, transcription of repetitive elements can trigger innate immune signaling. A recent study involving fallopian tube premalignant lesions and experimental ovarian cancer models demonstrated that loss of p53 leads to increased transcription of immunogenic repetitive elements and induces chronic viral-mimicry signaling. Rather than promoting sustained tumor elimination, this chronic exposure drove the selection of cells with enhanced tolerance to cytosolic nucleic acids and reduced immunogenicity, suggesting a potential mechanism for early immune evasion (Ishak et al., 2025).

TE derepression may affect both genome instability and transcriptional or immune programs. In high-grade serous ovarian carcinoma, repetitive-element activation may thus represent a convergence point of genomic instability, tumor evolution, and cellular adaptation.

## 5 ecDNA and complex structural variation: genome architecture beyond chromosomes

HGSOC genomes harbor overlapping classes of complex structural variation. In the 324-case deep whole-genome sequencing cohort, tumors associated with whole-genome duplication were enriched for chromothripsis, BFB events, and ecDNA, with substantial overlap observed among these distinct structural variant classes (Ewing et al., 2025). Approximately 16% of tumors harbored predicted ecDNA, a subset of which carried amplified oncogenes. At the *CCNE1* locus, diverse forms of complex structural alteration—including BFB, chromothripsis, and ecDNA—converged on high-level amplification, illustrating how different genome instability processes can give rise to similar oncogenic outcomes.

Extrachromosomal circular DNA spans a broad size range, and precise terminology is essential. Small extrachromosomal circular DNAs (eccDNAs) are commonly found in both normal and malignant cells, whereas the term ecDNA in contemporary cancer genomics typically refers to larger circular structures—often hundreds of kilobases to megabases in size—capable of carrying amplified genes and regulatory elements (Yan et al., 2024). Because ecDNAs lack centromeres, they can segregate unevenly during mitosis, enabling rapid copy-number heterogeneity and adaptive evolution under selective pressure (Bailey et al., 2024; Yan et al., 2024).

An early Circle-seq study in HGSOC identified abundant eccDNAs in four paired primary and metastatic tumors and revealed differences between the two disease sites (Cen et al., 2022). However, short-read approaches are inherently limited in reconstructing large, structurally complex circular amplicons. A recent study by Sun et al. addressed this challenge using long-read whole-genome sequencing of primary and paired metastatic tumors from 12 HGSOC patients, alongside six normal fallopian tube tissues. Their analyses revealed that ecDNA is more genomically unstable and less methylated than chromosomal DNA (Sun et al., 2025).

Importantly, the functional impact of ecDNA extended beyond oncogene copy number. HGSOC ecDNAs carried immunomodulatory genes and extensive enhancer sequences. Circular rearrangements enabled enhancers to engage noncanonical targets via enhancer hijacking and mobile-enhancer mechanisms, potentially amplifying transcriptional output. Even more strikingly, transposable elements were activated on hypomethylated ecDNA, and this activity correlated with epithelial–mesenchymal transition-related gene expression and adverse clinical outcomes (Sun et al., 2025).

These findings provide a conceptual link between processes often studied separately. Epigenetic instability can derepress repetitive elements, while structural rearrangements can generate ecDNA. In HGSOC, hypomethylated ecDNA has been associated with transposable element activation and altered enhancer activity. These observations raise the possibility that epigenetic dysregulation, transposable element activity, and structural variation may converge on individual ecDNA molecules. However, whether these features consistently co-occur and are functionally linked remains unclear. Long-read sequencing may help clarify their structural and epigenetic relationships, although further functional validation is needed (Li et al., 2025; Sun et al., 2025).

## 6 Discussion

Although *TP53* mutations, HRD, copy-number changes, and rearrangements define the conventional HGSOC framework, long-read sequencing and complete references reveal additional genomic complexity. Emerging evidence points to alterations in centromeres, telomeres, transposable elements, ecDNA, and other structurally intricate genomic regions, uncovering an additional layer of genome architecture that previous technologies could not fully resolve.

Beyond established non-coding regulators such as microRNAs, this review focuses on repetitive and structurally complex DNA. Repetitive elements, the epigenome, ecDNA, and chromosome architecture may interact rather than evolve independently. Dysfunction at centromeres and telomeres may contribute to chromosomal instability, while derepression of transposable elements may foster structural variation and transcriptional rewiring. ecDNA can simultaneously influence gene dosage, enhancer organization, DNA methylation, and repetitive-element activity. Nevertheless, several aspects of this model remain debated or unresolved. It is still unclear whether abnormalities in the repetitive genome act as drivers of genomic instability in high-grade serous ovarian carcinoma or are merely consequences of broader epigenetic dysregulation. Similarly, transposable-element activation may exert context-dependent effects, ranging from genome destabilization and viral-mimicry signaling to chronic immune adaptation. Whether ecDNA directly promotes transposable-element activation, or whether both phenomena stem from a shared hypomethylated state, also remains an open question.

These emerging features hold potential translational value, though most remain at the investigational stage. Among the more advanced TE-related biomarker candidates is ORF1p, which can be detected in HGSOC tissue, ascites, and plasma (Sato et al., 2023). Ultrasensitive plasma-based assays have also shown promising results for cancer detection, including ovarian cancer (Taylor et al., 2023). Other potential biomarkers include tumor-specific L1 insertions, ecDNA-derived sequences, centromeric or telomeric methylation patterns, and long-read haplotype-resolved epigenetic signatures (Li et al., 2025; Sun et al., 2025; Takamatsu et al., 2025; Pradhan et al., 2026). Clinical applicability remains unproven. Evidence from primary tumors, cell lines, and experimental models differs in biological context; short-read, long-read, and targeted assays also differ in sensitivity and ascertainment. Small cohorts, tumor purity, tissue-specific methylation, heterogeneity, and assay reproducibility further limit direct cross-study comparisons.

Long-read sequencing also has technical limitations. At typical genome-wide depth, low-frequency subclonal events may be missed, particularly in heterogeneous tumors. Many workflows require substantial amounts of intact, high-molecular-weight DNA, restricting use with small or archival FFPE specimens. Cancer-specific callers for complex structural variants and ecDNA reconstruction remain immature, and putative circular amplicons may require orthogonal validation. Even with T2T references, highly repetitive regions remain vulnerable to ambiguous mapping and reference bias (Li et al., 2025).

The same underlying biology may also expose therapeutic vulnerabilities. Active retrotransposition may generate DNA damage and replication stress, suggesting that tumors with high L1 activity could exhibit distinct dependencies on DNA damage response pathways (Mendez-Dorantes and Burns, 2023; Pradhan et al., 2026). Similarly, ecDNA-positive cancers may depend on mechanisms that support DNA replication, maintenance of circular DNA, and transcriptional amplification (Bailey et al., 2024; Yan et al., 2024). Epigenetic modulation of transposable elements could enhance viral mimicry, although chronic activation of repetitive elements may also foster immune tolerance (Ishak et al., 2025). These possibilities should therefore be viewed as mechanistic hypotheses rather than established therapeutic strategies.

Future priorities include larger HGSOC long-read cohorts, tissue-matched controls, and sampling of fallopian tube epithelium, p53 signatures, STICs, and invasive HGSOC. It will also be important to assess whether HRD- and WGD-dominated tumors acquire distinct repetitive-genome states, and whether long-read approaches can be reliably applied to archival FFPE samples and minute precursor lesions. Such efforts may help clarify the temporal ordering and functional relevance of these genomic abnormalities, and determine whether they can ultimately inform diagnosis, risk stratification, or treatment decisions.

Long-read sequencing offers new opportunities to investigate repetitive and structurally complex genomic regions that have remained difficult to characterize in HGSOC. Despite these advances, HGSOC-specific genome-wide long-read evidence remains largely limited to two small studies involving six patients (Takamatsu et al., 2025) and 12 patients (Sun et al., 2025). Larger, independent cohorts and functional studies are needed to assess the generalizability of these findings and determine the biological and potential clinical significance of the proposed relationships.
